# Knowledge, information needs and behavior regarding HIV and sexually transmitted infections among migrants from sub-Saharan Africa living in Germany: Results of a participatory health research survey

**DOI:** 10.1371/journal.pone.0227178

**Published:** 2020-01-27

**Authors:** Carmen Koschollek, Anna Kuehne, Johanna Müllerschön, Stephen Amoah, Helene Batemona-Abeke, Taty Dela Bursi, Pierre Mayamba, Adama Thorlie, Christina Mputu Tshibadi, Virginia Wangare Greiner, Viviane Bremer, Claudia Santos-Hövener

**Affiliations:** 1 Robert Koch Institute, Department for Infectious Disease Epidemiology, Unit for HIV/AIDS, STI and Blood-borne Infections, Berlin, Germany; 2 Charité University Medicine, Berlin, Germany; 3 Afrikaherz Berlin, Verband für interkulturelle Arbeit, Regionalverband Berlin/Brandenburg e.V., Berlin, Germany; 4 Pamoja Afrika e.V., Cologne, Germany; 5 Hannöversche Aids-Hilfe e.V., Hanover, Germany; 6 Aidshilfe Essen e.V., Essen, Germany; 7 Münchner Aids-Hilfe e.V., Munich, Germany; 8 Maisha e.V., Frankfurt am Main, Germany; University of the Witwatersrand, SOUTH AFRICA

## Abstract

**Background:**

A total of 3,419 new HIV diagnoses were reported in Germany in 2016, with migrants from sub-Saharan Africa (misSA) accounting for 14.1%. To understand the driving factors behind the epidemiological situation, we conducted a quantitative cross-sectional survey on knowledge, attitudes, behavior, and practices regarding HIV and sexually transmitted infections (STIs) among misSA living in six German cities utilizing participatory health research.

**Methods:**

Participants were recruited by peer researchers. Levels of knowledge, information needs, and preferred methods of information dissemination were analyzed to inform future prevention planning. Additionally, we analyzed sexual behavior and other risk factors for contracting HIV and STIs. The results may facilitate the formulation of targeted prevention messages in the future.

**Results:**

We included 2,432 participants in the analysis. General knowledge about HIV was adequate, as 86.9% were aware of the presented information. Statements about HIV co-infections were prior knowledge for 53.4% of the participants and about German HIV policies and HIV testing for 54.7%. Knowledge about other STIs differed, ranging from 69.6% who have ever heard of gonorrhea to 23.8% who have ever heard of genital warts. Groups with particular knowledge gaps were i) younger misSA, ii) recent migrants, iii) misSA without regular access to the German health care system, iv) misSA of lower socioeconomic status, and v) misSA with Muslim religious affiliation. The majority of participants reported information needs (72.8%), and 71.3% wanted to obtain this information from health professionals. Male misSA were more likely to report five or more sexual partners compared to females. Less than half of participants reported always using condoms with non-steady sexual partners (46.8%). Reasons for not using condoms differed between males and females. A considerable proportion of females (16.3%) and males (6.8%) experienced sexualized violence. More than one fourth of women (26.9%) were affected by female genital mutilation/cutting.

**Discussion:**

Future prevention planning should focus on sub-groups with particular knowledge gaps, recognizing their preferred methods of information dissemination. Prevention messages for male misSA should focus on their own risk perception and for female misSA on empowerment, e.g. to negotiate condom use.

## Background

### HIV and STIs among migrants from sub-Saharan Africa living in Germany

Globally, 36.7 million people were living with HIV in 2016 according to UNAIDS; two in three of these people were living in sub-Saharan Africa (69.5%) [1]. In the same year, 14.4% of reported cases from 30 EU/EEA countries originated from sub-Saharan Africa [2].

In Germany, 3,419 new HIV diagnoses were reported to the Robert Koch Institute (RKI) in 2016, and 3,235 notifications included information on country of origin; of these, 14.9% were migrants from sub-Saharan Africa (n = 483) [3]. The HIV epidemic in Germany is concentrated within sub-populations, mostly affecting men having sex with men. However, the transmission route for every fourth diagnosis in 2016 was heterosexual transmission, and among these 51.8% originated from sub-Saharan Africa [3]. The majority of these cases acquired the infection in sub-Saharan Africa (68.5%), whereas 6.3% of the infections were most likely contracted in Germany [3]. However, the literature suggests substantial underreporting of the host country as the country of HIV infection [4–8]. It was estimated that 88,400 people were living with HIV in Germany in 2016, including 6,400 migrants from sub-Saharan Africa (7.2%) who acquired their infection outside of Germany [9]. Between 2014 and 2016, 789 AIDS cases were voluntarily reported to the RKI by medical HIV specialists in Germany [3]. Among these, 13% of cases originated from sub-Saharan Africa [3]. Although the presented data on HIV is limited, as no baseline data regarding HIV tests conducted within a year exist, the data suggest a high vulnerability of migrants from sub-Saharan Africa for contracting HIV abroad but also in Germany [3, 10].

Estimates on the incidence or prevalence of sexually transmitted infections (STIs) among migrants from sub-Saharan Africa in Germany do not exist. Migrants from sub-Saharan Africa accounted for only a small proportion of 0.3% of reported cases of syphilis in Germany between 2002 and 2013 [11].

Migrants with a citizenship of a sub-Saharan African country represent only a small proportion of 0.4% of the population in Germany [12,13]. Even though this figure does not include migrants originating from sub-Saharan Africa with German citizenship or with an undocumented legal status, the ratio of HIV diagnoses or people living with HIV and population size is much higher than that of the general population. Therefore, migrants from sub-Saharan Africa are disproportionately affected by HIV in Germany, and surveillance and prevention in this population are particularly important.

### From behavioral surveillance to community-based participatory health research

Surveillance data as well as previous research suggest that migrants from sub-Saharan Africa in Germany are more likely to be diagnosed and treated at a later stage of HIV than persons of German origin [2, 8, 14]. Lack of knowledge about transmission routes or limited access to health services as well as fear and stigma might play a role for ongoing HIV transmissions and might be, next to others, reasons for late presentation. The World Health Organization (WHO) and the European Centre for Disease Prevention and Control (ECDC) recommend complementing routine HIV and STI surveillance data with indicator-based data on behavior and practices to understand the driving factors behind the epidemiological situation [15–19].

Surveys on knowledge, attitudes, behaviors, and practices (KABP) regarding HIV and STIs for the general population do not include migrants from sub-Saharan Africa sufficiently [20]. Other European countries such as the UK facing the same challenges started to actively involve the target population in planning and conducting the study within population based surveys [21–23], as recommended by the WHO and the ECDC [16, 17]. Community-based participatory health research (CBPHR) involves community members from the beginning of study planning, during study implementation up to interpretation of results, as well as in the development of recommendations [24–29]. So far in Germany, only small CBPHR surveys among migrants from sub-Saharan Africa have been carried out on the local level [24, 30, 31].

Thus, we utilized CBPHR, involving members of African communities; stakeholders in HIV prevention, counseling and testing; and researchers in the fields of HIV for the development and piloting of the study design. Both are described elsewhere [32, 33]. In 2014, we started the cross-sectional, full-scale study to identify KABP regarding HIV and STIs among migrants from sub-Saharan Africa in six German cities and regions to facilitate the development of targeted interventions for African communities in Germany.

### Objectives

Within this paper, we aim i) to describe the level of knowledge about HIV and STIs among migrants from sub-Saharan Africa living in Germany and ii) to determine sub-groups with particular knowledge gaps. Furthermore, we describe iii) information needs and preferred methods of information dissemination in order to inform future prevention planning. Additionally, we aim iv) to describe behavioral patterns and other risk factors, that is, sexualized violence and female genital mutilation/cutting (FGM/C), that are associated with higher risks of contracting HIV and STIs, in order to facilitate the formulation of targeted prevention messages for migrants from sub-Saharan Africa living in Germany.

## Methodology

### Study design and sampling procedures

We conducted a cross-sectional survey among migrants from sub-Saharan Africa living in Germany on KABP regarding HIV, viral hepatitis (HEP), and other STIs. Participants were recruited by trained peer researchers, that is, members of local African communities, in six German cities and regions. Study cities were chosen according to the number of migrants with citizenship of a sub-Saharan African country who were officially registered in the respective city or region at the end of 2013 according to foreigners’ statistics, including Munich, the Rhine-Ruhr region, Cologne, Berlin, Frankfurt am Main, and the region of Hanover. In every city, we collaborated with a local partner organization that was responsible for research operations, that is, recruiting of a local study coordinator and peer researchers as well as the organization of different meetings for process evaluation.

Data collection took place successively in the different study cities between January 2015 and May 2016. To carry out data collection, the partner organization, the RKI, and peer researchers from either the pilot study or from former study cities trained peer researchers of the subsequent study city in a two-day training on research ethics, study goals, the questionnaire, HIV, HEP, STIs, and recruitment strategies. After training, the peer researchers started recruitment at locations that were identified in community mapping during the training (e.g., churches, mosques, shops, associations, sports clubs) using convenience sampling. After obtaining verbal consent by participants, they either handed out the paper-based questionnaires for self-completion or carried out face-to-face or telephone interviews. Participants chose their preferred mode of administration; peer researchers used the same questionnaire for conducting an interview or self-completion. Questionnaires were available in German, English, and French; in addition, peer researchers were proficient in several African languages, such as Swahili, Lingala, or Twi. Questionnaires were sent back to the RKI in post-paid envelopes. We compared gender and countries of birth of recruited participants to data of official foreigners’ statistics [34] on a weekly basis and provided weekly feedback to the partner organization and peer researchers. This was done to steer recruitment in order to firstly reach the larger communities regarding countries of origin and an appropriate gender ratio corresponding to official statistics and secondly to create heterogeneity regarding other sociodemographic characteristics, such as in terms of different religious affiliations or different health insurance statuses. We made rigorous attempts to reach a heterogeneous sample, and encouraged peer researchers to recruit more people of possibly underrepresented groups. Details on the study design and sampling can be found elsewhere [35, 36]. Full ethical clearance was granted by the Ethical Committee at the medical school of Charité, Berlin, in November 2014 (EA4/105/14).

### Questionnaire and measurements

The questionnaire included indicators for behavioral surveillance in migrant and ethnic minority populations recommended by the ECDC [17]. It consisted of questions on sociodemographic characteristics; knowledge about HIV, HEP, and STIs; testing for these infections; sexual behavior and other risk factors in terms of contracting HIV, HEP, and STIs; behavior towards people living with HIV and self-reported information needs; and preferred methods of information dissemination (see [35] for a full list of indicators). The questions were partially adapted from a survey used within the BASS-Line study in the UK with migrants from sub-Saharan Africa [22] and partially developed by the working group. Within this paper, we focus on knowledge about HIV and STIs, including self-reported information needs and preferred methods of information dissemination, as well as on sexual behavior and other risk factors, as presented in Table 1.

**Table 1:** Indicators covered within the questionnaire of the misSA study in Germany, 2014–2016 and analyzed in the present study.

Sociodemographic characteristics		
	Sex, age, country of birth (participant and parents), length of stay in Germany, German language proficiency, educational level, monthly net income, religious affiliation, health insurance status	
Knowledge about HIV and STIs per section—-—*"Did you know this before*?*"*		
General knowledge about HIV		
	HIV and Aids also exist in Germany.	
	Aids is caused by a virus called HIV.	
	You cannot tell from someone’s appearance whether he or she has HIV or not.	
	There is a test which shows whether someone is HIV positive or not.	
	HIV is not transmitted through kissing or shaking hands.	
	HIV can be transmitted through sexual intercourse.	
	There is no cure for HIV infection.	
	There are medications that can help people with HIV stay healthy.	
Knowledge about HIV co-infections		
	People that have sexually transmitted infections have an increased risk of contracting HIV.	
	People with HIV have an increased risk of contracting tuberculosis.	
Knowledge about German HIV policies and HIV testing		
	Africans are not deported from Germany just for having HIV.	
	In [study city] you can get tested for HIV anonymously and for free, e.g. at the local Public Health Department.	
Knowledge about STIs		
	Gonorrhea	
	Syphilis	
	Herpes	
	Genital warts	
	Chlamydia	
Self-reported information needs—-—*"What topics would you like to have more information on*?*"*		
	The risk of infection/transmission of HIV	
	The risk of infection/transmission of hepatitis B and C	
	The risk of infection/transmission of other STIs	
	Transmission risks of tuberculosis	
	How can I protect myself (against HIV and STIs	
	Test and diagnostic options	
	Medical treatment (for HIV and hepatitis)	
	On female circumcision *(topic was not offered in the first study city Munich)*	
	Support for patients with HIV	
	Support for family members	
Methods of information dissemination, grouped—-—*"How would you like to get more information on these topics*?*"*		
Health professionals		
	From a counseling center	
	From medical professionals	
Personal environment		
	From someone from my community	
	From friends	
Classic and print media		
	From radio and TV	
	From flyers and brochures	
	From African magazines *(not offered in the first study city Munich)*	
New media		
	From websites	
	Through mobile/smart phones	
	From social networks on the Internet	
Workshops		
	Through participation in a workshop	
Sexual behavior and other risk factors		
Sexual behavior		
	Age at first sexual intercourse	
	Sexual attraction	
	Sex within the last 12 months	
	Number of male and female sexual partners within the last 12 months, categorical	
	Sex with steady/permanent sexual partner(s) within the last 12 months	
	Origin of steady/permanent sexual partner(s)	
	Sex with non-steady sexual partner(s) within the last 12 months	
	Condom use with non-steady sexual partner(s)	
	Condom use at last sexual intercourse	
	Reasons for not using condoms	
Other risk factors		
	Lifetime experience of sexualized violence (once or repeated)	
	Female genital mutilation/cutting (FGM/C)	

Knowledge about HIV was determined by presenting true statements and participants were asked if they knew the information before (e.g. “Aids is caused by a virus called HIV” with the answer categories “I knew this already”, “I was not sure if that was true or not”, “I didn’t know this”, and “I do not understand this statement”). Knowledge about other STIs was ascertained by the question “Which of the sexually transmitted diseases have you heard about apart from HIV?”.

### Data analysis

All data were entered into the VOXCO interviewer web™, an online survey and data collection software. Data entry was checked by a second person at the RKI. After data cleaning, data analysis was performed using Stata version 14.

The calculated sample size for all six study cities and regions was 3,009 participants; calculations are described elsewhere [35].

Questionnaires were eligible for inclusion in the study if there were no missing or ambiguous answers regarding sex, whether at least one of the participants’ parents was born in a sub-Saharan African country, and at least 60% of the questionnaire was filled in.

Questionnaires were excluded from analysis for all participants i) not born in a sub-Saharan African country or with missing or ambiguous answers regarding their country of birth, as well as ii) those reporting living in Germany since birth or with missing or ambiguous answers regarding their length of stay in Germany. We also excluded iii) participants with missing or ambiguous answers in at least one of the categories of age or education.

For the descriptive analysis of the study population, we used chi-squared tests to examine differences between males and females in terms of sociodemographic characteristics. Differences in medians were analysed using Mann Whitney-U tests.

To analyze knowledge about HIV, statements were summarized in three sections–general knowledge about HIV, knowledge about co-infections, and knowledge about German HIV policies and HIV testing–as displayed in Table 1. The five STIs were also summarized and are reported under the section regarding knowledge about STIs. In the following analyses, we calculated the aggregated number of positive answers (“I knew this already” vs. all other answers for HIV and “I have already heard of this STI” vs. all other answers) per section. We used a logistic two-level model with random effects, that is, participants on the first level and their answers on the second level. Models for multivariable analysis (MVA) were developed using forward regression and thus stepwise integration of characteristics that proved to be significant in univariable analysis (UVA) using a significance level of p ≤ 0.05. Adjusted odds ratios (aOR) and 95% confidence intervals (CI) were calculated. We included gender next to (sociodemographic) variables that were significant in UVA in all four sections: age, educational level, length of stay in Germany, German language proficiency, monthly net income, health insurance status and religious affiliation.

Self-reported information needs were analyzed descriptively. Preferred methods of information dissemination were grouped as displayed in Table 1. To detect differences between sub-groups, UVA was performed; unadjusted odds ratios (OR) and 95% CI were calculated.

Indicators describing sexual behavior and sexualized violence were stratified by sex. The numbers of male and female sexual partners were aggregated to the total number of sexual partners. Unadjusted OR and 95% CI were calculated to examine differences in behavioral and risk patterns between males and females. Within the group of women, we stratified FGM/C by age, educational level, length of stay in Germany, health insurance status, and religious affiliation, and we calculated unadjusted OR and 95% CI to identify factors associated with FGM/C.

## Results

### Study population

We received 3,178 questionnaires at the RKI, of which 3,040 were eligible for inclusion in the study; 48 were excluded due to incompleteness, and in 90 questionnaires there was missing information on sex and/or the sub-Saharan African country of birth of at least one parent. After excluding participants for their i) country of birth (n = 329), ii) length of stay in Germany (n = 67), and iii) age and/or education (n = 212) as described above, the final sample size was 2,432 participants.

### Sociodemographic characteristics

Of the total sample, 45.5% were female, the median age was 32 years, and the median length of stay in Germany was nine years. Females and males differed markedly in terms of age, educational level, length of stay in Germany, monthly net income, health insurance status, and religious affiliation (Table 2). Good or very good German language proficiency was reported by 47.8% of the participants. Participants were born in 43 different sub-Saharan African countries; the main countries of birth were Ghana (13.9%), Nigeria (13.6%), and Cameroon (11.7%).

**Table 2:** Sociodemographic characteristics of male and female participants of the misSA study in Germany, 2014–2016, χ^2^ test, n = 2,432.

		Female (n = 1,106)		Male (n = 1,326)		p-value
		n	%	n	%	
Age						
	Median age in years (range)	32 (18–78)		33 (18–77)		**0.012**
	18–25 years	235	21.3%	300	22.6%	**<0.001**
	26–35 years	440	39.8%	453	34.2%	
	36–45 years	297	26.9%	301	22.7%	
	> 45 years	134	12.1%	272	20.5%	
Educational level						
	No school/primary or secondary school	448	40.5%	436	32.9%	**<0.001**
	High school/vocational school	374	33.8%	419	31.5%	
	University/college	284	25.7%	473	35.7%	
Length of stay in Germany						
	Median length of stay in months (range)	79 (1–504)		60 (1–540)		**0.012**
	< 5 years	431	39.0%	638	48.1%	**<0.001**
	≥ 5 years	675	61.0%	688	51.9%	
German language proficiency						
	No or little	307	27.8%	386	29.11%	0.824
	Average or good	523	47.3%	613	46.2%	
	Very good or mother tongue	268	24.2%	320	24.1%	
	Unknown	8	0.7%	7	0.5%	
Monthly net Income						
	< 1,000 Euro per month	592	53.5%	592	44.7%	**<0.001**
	≥ 1,000 Euro per month	258	23.3%	432	32.6%	
	Unknown	256	23.2%	302	22.8%	
Health insurance status						
	Regular health insurance	916	82.8%	1,028	77.5%	**0.005**
	No health insurance or medical treatment voucher for asylum seekers from social welfare office	166	15.0%	260	19.6%	
	Unknown	24	2.2%	38	2.9%	
Religious affiliation						
	Christian	792	71.6%	814	61.4%	**<0.001**
	Muslim	254	23.0%	388	29.3%	
	No, other, or unknown religion	60	5.4%	124	9.4%	

### Knowledge about HIV

Of all the participants, 86.9% had knowledge of the eight general statements on HIV. The least known was the information that there is no cure for HIV infection (80.7%), while the information that HIV can be transmitted through sexual intercourse was known most frequently (94.9%). MVA showed associations between general knowledge about HIV and health insurance status, age, educational level, German language proficiency, religious affiliation, monthly net income, and length of stay in Germany (Table 3).

**Table 3:** Uni- and multivariable analyses for the association between knowledge about HIV in general and HIV co-infections per section and sociodemographic characteristics of the participants of the misSA study in Germany, 2014–2016, questions answered by 2,432 participants*.

Knowledge section		HIV general							HIV co-infections						
Variable			Univariable analysis			Multivariable analysis				Univariable analysis			Multivariable analysis		
		%	OR	95%—CI	p-value	aOR	95%—CI	p-value	%	OR	95%—CI	p-value	aOR	95%—CI	p-value
Sex															
	Men	86.3%	Ref.			Ref.			54.9%	Ref.			Ref.		
	Women	87.6%	1.17	0.97–1.40	0.100	**1.23**	**1.03**–**1.47**	**0.024**	51.6%	**0.81**	**0.66**–**0.999**	**0.050**	0.89	0.72–1.09	0.251
Age															
	18–25 years	81.0%	**0.43**	**0.33**–**0.56**	**<0.001**	**0.59**	**0.45**–**0.78**	**<0.001**	43.3%	**0.38**	**0.28**–**0.51**	**<0.001**	**0.43**	**0.32**–**0.60**	**<0.001**
	26–35 years	87.9%	0.93	0.73–1.18	0.565	1.00	0.79–1.27	0.984	55.6%	0.82	0.63–1.08	0.161	**0.75**	**0.57**–**0.98**	**0.034**
	36–45 years	89.1%	Ref.			Ref.			58.6%	Ref.			Ref.		
	> 45 years	89.2%	0.97	0.72–1.30	0.827	0.91	0.68–1.20	0.488	54.2%	0.75	0.54–1.04	0.087	**0.68**	**0.49**–**0.93**	**0.016**
Educational level															
	No school/primary or secondary school	80.1%	**0.43**	**0.35**–**0.53**	**<0.001**	**0.54**	**0.44**–**0.66**	**<0.001**	38.6%	**0.40**	**0.31**–**0.50**	**<0.001**	**0.48**	**0.38**–**0.62**	**<0.001**
	High school/vocational school	88.5%	Ref.			Ref.			53.9%	Ref.			Ref.		
	University/college	93.1%	**2.15**	**1.70**–**2.73**	**<0.001**	**1.94**	**1.53**–**2.46**	**<0.001**	70.2%	**2.81**	**2.17**–**3.64**	**<0.001**	**2.32**	**1.79**–**2.99**	**<0.001**
Length of stay in Germany															
	< 5 years	83.7%	**0.53**	**0.44**–**0.63**	**<0.001**	1.06	0.83–1.35	0.637	50.3%	**0.70**	**0.57**–**0.87**	**0.001**	**1.45**	**1.10**–**1.91**	**0.008**
	≥ 5 years	89.4%	Ref.			Ref.			55.8%	Ref.			Ref.		
German language proficiency															
	No, little, or unknown	81.0%	**0.44**	**0.36**–**0.54**	**<0.001**	**0.68**	**0.54**–**0.86**	**0.001**	44.2%	**0.55**	**0.43**–**0.70**	**<0.001**	**0.70**	**0.54**–**0.92**	**0.011**
	Average or good	88.7%	Ref.			Ref.			53.7%	Ref.			Ref.		
	Very good or mother tongue	90.3%	**1.36**	**1.07**–**1.72**	**0.012**	1.09	0.87–1.38	0.452	63.9%	**1.93**	**1.48**–**2.52**	**<0.001**	**1.61**	**1.24**–**2.09**	**<0.001**
Monthly net income															
	< 1,000 Euro per month	85.3%	**0.49**	**0.39**–**0.61**	**<0.001**	0.84	0.66–1.07	0.160	50.2%	**0.54**	**0.42**–**0.69**	**<0.001**	0.90	0.69–1.17	0.433
	≥ 1,000 Euro per month	90.9%	Ref.			Ref.			59.9%	Ref.			Ref.		
	Unknown	85.2%	**0.46**	**0.36**–**0.60**	**<0.001**	**0.68**	**0.53**–**0.88**	**0.004**	52.2%	**0.61**	**0.45**–**0.82**	**0.001**	0.85	0.64–1.14	0.280
Health insurance status															
	Regular health insurance	88.9%	Ref.			Ref.			56.2%	Ref.			Ref.		
	No health insurance or medical treatment voucher for asylum seekers or unknown	78.8%	**0.35**	**0.28**–**0.43**	**<0.001**	**0.70**	**0.55**–**0.89**	**0.003**	42.3%	**0.41**	**0.32**–**0.54**	**<0.001**	0.83	0.62–1.11	0.215
Religious affiliation															
	Christian	88.9%	Ref.			Ref.			58.1%	Ref.			Ref.		
	Muslim	81.8%	**0.46**	**0.38**–**0.57**	**<0.001**	**0.70**	**0.57**–**0.85**	**<0.001**	42.4%	**0.37**	**0.29**–**0.47**	**<0.001**	**0.54**	**0.42**–**0.68**	**<0.001**
	No, other, or unknown religion	87.0%	0.78	0.56–1.11	0.165	0.87	0.63–1.21	0.414	50.5%	**0.62**	**0.42**–**0.92**	**0.017**	0.70	0.48–1.02	0.065

Both statements on HIV co-infections were known by 53.4% of the participants. Of all the participants, 55.6% knew that people with HIV have an increased risk of contracting tuberculosis, and 52.2% knew that people who have an STI have an increased risk of contracting HIV. In MVA, age, educational level, religious affiliation, German language proficiency, and length of stay in Germany were associated with knowledge about HIV co-infections (Table 3).

Both statements about German HIV policies and HIV testing were known by 54.7% of the participants. Of all the participants, 56.2% knew that Africans cannot be deported from Germany just for having HIV, and 53.2% knew that one can get tested for HIV anonymously and for free at the local Public Health Departments. Factors associated with knowledge about German HIV policies and HIV testing in MVA were length of stay in Germany, age, religious affiliation, educational level, and German language proficiency (Table 4).

**Table 4:** Uni- and multivariable analyses for the association between knowledge about German HIV policies and HIV testing and STIs per section and sociodemographic characteristics of participants of the misSA study in Germany, 2014–2016, questions answered by 2,432 participants* (continued).

Knowledge section		German HIV policies and HIV testing							STIs						
Variable			Univariable analysis			Multivariable analysis				Univariable analysis			Multivariable analysis		
		%	OR	95%—CI	p-value	aOR	95%—CI	p-value	%	OR	95%—CI	p-value	aOR	95%—CI	p-value
Sex															
	Men	54.8%	Ref.			Ref.			43.1%	Ref.			Ref.		
	Women	54.5%	0.98	0.81–1.19	0.844	0.95	0.78–1.15	0.569	45.6%	1.13	0.999–1.29	0,051	**1.22**	**1.08**–**1.37**	**0.001**
Age															
	18–25 years	42.5%	**0.36**	**0.27**–**0.48**	**<0.001**	**0.60**	**0.45**–**0.81**	**0.001**	34.5%	**0.49**	**0.40**–**0.59**	**<0,001**	**0.63**	**0.52**–**0.75**	**<0.001**
	26–35 years	53.9%	**0.70**	**0.55**–**0.90**	**0.005**	0.86	0.68–1.11	0.244	47.1%	0.96	0.82–1.13	0,632	0.97	0.83–1.12	0.641
	36–45 years	60.0%	Ref.			Ref.			47.8%	Ref.			Ref.		
	> 45 years	64.7%	1.34	0.98–1.81	0.063	1.07	0.80–1.44	0.661	45.7%	0.90	0.74–1.09	0,280	0.86	0.72–1.02	0.087
Educational level															
	No school/primary or secondary school	45.3%	**0.50**	**0.40**–**0.63**	**<0.001**	**0.69**	**0.55**–**0.86**	**0.001**	30.5%	**0.48**	**0.42**–**0.56**	**<0,001**	**0.57**	**0.50**–**0.66**	**<0.001**
	High school/vocational school	57.1%	Ref.			Ref.			44.6%	Ref.			Ref.		
	University/college	63.1%	**1.43**	**1.13**–**1.82**	**0.003**	**1.39**	**1.10**–**1.76**	**0.007**	60.0%	**2.14**	**1.85**–**2.47**	**<0,001**	**1.98**	**1.72**–**2.28**	**<0.001**
Length of stay in Germany															
	< 5 years	42.9%	**0.29**	**0.24**–**0.36**	**<0.001**	**0.54**	**0.42**–**0.70**	**<0.001**	38.9%	**0.61**	**0.54**–**0.69**	**<0,001**	1.01	0.87–1.18	0.885
	≥ 5 years	63.9%	Ref.			Ref.			48.4%	Ref.			Ref.		
German language proficiency															
	No, little, or unknown	40.8%	**0.39**	**0.31**–**0.49**	**<0.001**	**0.72**	**0.56**–**0.92**	**0.009**	35.0%	**0.59**	**0.51**–**0.69**	**<0,001**	0.88	0.76–1.03	0.108
	Average or good	57.0%	Ref.			Ref.			44.9%	Ref.			Ref.		
	Very good or mother tongue	67.0%	**1.84**	**1.45**–**2.34**	**<0.001**	**1.39**	**1.09**–**1.77**	**0.008**	54.0%	**1.58**	**1.36**–**1.85**	**<0,001**	**1.28**	**1.10**–**1.48**	**0.001**
Monthly net income															
	< 1,000 Euro per month	49.2%	**0.40**	**0.32**–**0.50**	**<0.001**	0.91	0.71–1.16	0.437	41.1%	**0.59**	**0.51**–**0.68**	**<0,001**	0.90	0.78–1.05	0.186
	≥ 1,000 Euro per month	64.6%	Ref.			Ref.			51.4%	Ref.			Ref.		
	Unknown	45.8%	**0.54**	**0.41**–**0.70**	**<0.001**	0.96	0.73–1.25	0.739	42.2%	**0.62**	**0.52**–**0.74**	**<0,001**	**0.85**	**0.72**–**0.998**	**0.048**
Health insurance status															
	Regular health insurance	58.6%	Ref.			Ref.			47.9%	Ref.			Ref.		
	No health insurance or medical treatment voucher for asylum seekers or unknown	39.2%	**0.32**	**0.25**–**0.41**	**<0.001**	0.80	0.61–1.05	0.101	29.6%	**0.38**	**0.32**–**0.44**	**<0,001**	**0.66**	**0.56**–**0.78**	**<0.001**
Religious affiliation															
	Christian	58.6%	Ref.			Ref.			47.9%	Ref.			Ref.		
	Muslim	45.6%	**0.46**	**0.37**–**0.58**	**<0.001**	**0.69**	**0.56**–**0.86**	**0.001**	35.2%	**0.51**	**0.45**–**0.60**	**<0,001**	**0.74**	**0.65**–**0.84**	**<0.001**
	No, other, or unknown religion	52.5%	**0.70**	**0.48**–**1.002**	**0.052**	**0.74**	**0.52**–**1.04**	**0.085**	44.4%	**0.84**	**0.66**–**1.07**	**0,154**	0.98	0.79–1.22	0.871

* Absolute numbers for each variable category are displayed in Table 2.

### Knowledge about STIs

While gonorrhoea (69.6%) and syphilis (65.8%) were known by the majority of the participants, herpes (37.0%), chlamydia (25.0%), and genital warts (23.8%) were less likely to be known. Overall, 14.4% of participants reported not knowing any of the presented STIs. In MVA, knowledge about STIs was associated with educational level, age, health insurance status, monthly net income, religious affiliation, sex, and German language proficiency (Table 4).

### Information needs and methods of information dissemination

Overall, 72.8% of participants indicated a need for more information about HIV and STIs (n = 1,771). Participants younger than 36 years of age were less likely to state information needs (18–25 vs. 36–45 years: 71.2% vs. 77.8%; OR = 0.71; 95% CI: 0.54–0.93 | 26–35 vs. 36–45 years: 72.2% vs. 77.8%; OR = 0.74; 95% CI: 0.58–0.94). Other sociodemographic factors showed no impact.

Among those who wanted more information about HIV and STIs 1,728 indicated at least one topic. Most relevant was the risk of transmission of HEP (66.7%), followed by the risk of transmission of HIV (56.3%) and the risk of transmission of other STIs (53.5%). Further relevant topics were means of protection against HIV and STIs (44.1%), transmission risks of tuberculosis (42.0%), and about medical treatment for HIV and HEP (40.2%). Information about options to support people living with HIV (33.2%), testing and diagnostic options (28.8%), FGM/C (24.3%), and options to support family members of people living with HIV (22.3%) were less often reported.

At least one preferred method of information dissemination was stated by 1,712 participants who wanted to know more about HIV and STIs. The majority wanted to receive the information from health professionals (71.3%), followed by participation in a workshop (50.6%) and new media (47.6%); new media was more likely to be stated by younger participants and those with higher educational levels. Classic and print media (40.0%) and obtaining more information from the personal environment (29.3%) were less often reported; obtaining information from the personal environment was more likely to be reported by participants with lower educational levels (for more details, see S1 Table).

### Sexual behavior and other risk factors

The majority of the participants reported having had sex, while 101 participants reported that they have never had sex before. Among those who have ever had sex, the majority also had sex within the last 12 months. Females were more likely to report sex with (one or more) steady sexual partner(s), while males more frequently reported having had sex with non-steady sexual partner(s); they were also more likely to report having had sex with steady and non-steady sexual partners (Table 5). Females were more likely to report having had sex with only one sexual partner, while males more frequently reported having had sex with more than five sexual partners within the last year (Table 5).

**Table 5:** Univariable analyses: Sexual behavior and other risk factors of male and female participants of the misSA study in Germany, 2014–2016.

		Female		Male		OR	95%-CI	p-value
		n	%	n	%			
**Sexual behavior**							
**Lifetime sexual activity**							
Ever had sex	1,020	95.2%	1,226	96.1%	0.82	0.55–1.22	0.317
Age at first sexual intercourse							
Under 16 years of age	157	15.4%	228	18.6%	**0.80**	**0.64**–**0.995**	**0.045**
Sexual attraction							
Attracted to both sexes or same sex	57	6.4%	44	3.9%	**1.70**	**1.13**–**2.54**	**0.010**
**Sexual activity within the last 12 month **							
Had sex within the last 12 months	812	79.8%	988	81.3%	0.92	0.74–1.13	0.406
Sexual partners							
Sex with steady sexual partner(s)	647	84.0%	704	74.6%	**1.79**	**1.41**–**2.27**	**<0.001**
Sex with non-steady sexual partner(s)	161	21.7%	335	37.3%	**0.47**	**0.37**–**0.58**	**<0.001**
Sex with steady and non-steady sexual partners	99	15.7%	216	31.7%	**0.40**	**0.31**–**0.53**	**<0.001**
Origin of steady sexual partner(s)							
Germany	179	27.9%	227	33.1%	**0.78**	**0.62**–**0.99**	**0.040**
Country of origin	387	60.3%	368	53.6%	**1.31**	**1.06**–**1.63**	**0.015**
Other countries	88	13.7%	116	16.9%	0.78	0.58–1.05	0.106
Number of sexual partners							
Single sexual partner	642	83.1%	601	63.7%	**2.79**	**2.22**–**3.51**	**<0.001**
More than five sexual partners	7	0.9%	37	3.9%	**0.22**	**0.10**–**0.51**	**<0.001**
**Condom use**							
Always with non-steady sexual partners	77	48.1%	153	46.8%	1.06	0.72–1.54	0.781
At last sexual intercourse	286	30.2%	522	45.8%	**0.51**	**0.43**–**0.61**	**<0.001**
Reasons for not using condoms°							
I am faithful to my partner.	294	32.4%	294	26.8%	**1.31**	**1.08**–**1.59**	**0.006**
I want to feel my partner.	161	17.8%	236	21.5%	**0.79**	**0.63**–**0.99**	**0.037**
My partner doesn't want us to use it.	164	18.1%	127	11.6%	**1.69**	**1.31**–**2.17**	**<0.001**
I don't like condoms.	116	12.8%	158	14.4%	0.87	0.68–1.13	0.303
I would like to get pregnant/ have a child with my partner.	173	19.1%	73	6.6%	**3.31**	**2.48**–**4.43**	**<0.001**
I find it embarrassing.	107	11.8%	122	11.1%	1.07	0.81–1.41	0.626
I always use a condom.	118	13.0%	268	24.4%	**0.46**	**0.37**–**0.59**	**<0.001**
**Other risk factors**							
**Sexualized violence**							
Experience of sexualized violence							
Once	103	10.0%	53	4.3%	**2.49**	**1.77**–**3.50**	**<0.001**
Repeated	65	6.3%	29	2.3%	**2.81**	**1.80**–**4.39**	**<0.001**
Place where sexualized violence was experienced*							
Germany	51	31.5%	39	48.8%	**0.48**	**0.29**–**0.84**	**0.009**
Country of origin	109	67.3%	32	40.0%	**3.09**	**1.77**–**5.37**	**<0.001**
Other country	26	16.1%	11	13.8%	1.20	0.56–2.57	0.640
**FGM/C among women**	**Proportion of women affected **						
Age group							
18–25 years	62	29.0%			1.04	0.70–1.54	0.844
26–35 years	108	25.8%			0.89	0.63–1.24	0.482
36–45 years	80	28.2%			Ref.		
> 45 years	31	24.4%			0.82	0.51–1.33	0.428
Educational level							
No school/primary or secondary school	144	33.9%			**1.43**	**1.05**–**1.95**	**0.025**
High school/vocational school	93	26.4%			Ref.		
University/ college	44	16.5%			**0.55**	**0.37**–**0.82**	**0.003**
Length of stay in Germany							
< 5 years	127	32.2%			**1.53**	**1.16**–**2.02**	**0.003**
≥ 5 years	154	23.7%			Ref.		
Health insurance status							
Regular health insurance	219	25.3%			Ref.		
No health insurance or medical treatment voucher for asylum seekers or unknown	62	35.0%			**1.60**	**1.13**–**2.25**	**0.008**
Religious affiliation							
Christian	158	20.8%			Ref.		
Muslim	111	47.4%			**3.44**	**2.52**–**4.70**	**<0.001**
No, other, or unknown religion	12	24.5%			1.24	0.63–2.43	0.535

° Multiple answers were possible; only answers mentioned by at least 10% of participants.

* Multiple answers were possible.

Of those who had sex with non-steady sexual partners in the last 12 months, less than half reported that they always used condoms. Among all participants who ever had sex, less than half of males and less than one third of females reported that they used a condom during their last intercourse (Table 5). Participants provided different reasons for not using condoms; females were more likely to state that they were faithful to their partner, their partner did not want to use condoms, or that they wanted to get pregnant. Males were more likely to state that they would not use condoms as they “wanted to feel their partner”. Males were more likely to state that they always use condoms (Table 5).

Sexualized violence was experienced by 16.3% of women (168/1,029) and 6.6% of males (82/1,237). Females most often had experienced sexualized violence in their country of origin, while males most often reported having experienced sexualized violence in Germany (Table 5).

FGM/C was reported by 26.9% of women (281/1,044). Women with Muslim religious affiliation, without regular health insurance, and with a shorter length of stay in Germany and lower educational levels were more likely to be affected (Table 5).

## Discussion

Utilizing community-based participatory health research allowed us to recruit a heterogeneous sample of migrants from sub-Saharan Africa in six cities and regions in Germany. A total of 45.5% of the sample were female, which reflected the official data of German foreigners’ statistics in 2013, which reports 46% females with a citizenship of a sub-Saharan African country [34]. The three main countries of birth within our sample were Ghana, Nigeria, and Cameroon and they made a proportion of 39.2%. According to the foreigners’ statistics, citizenships of these countries accounted for 35.8% of all migrants with a citizenship of a sub-Saharan African country in Germany [34].

We found adequate levels of knowledge about general statements on HIV and knowledge gaps regarding HIV co-infections and about German HIV policies and HIV testing. Knowledge gaps were also found regarding STIs; common STIs such as chlamydia and genital warts were only known by one fourth of participants. In line with these results, the majority of participants stated information needs about HIV and STIs. In addition, we found more sexual risk behavior among male compared to female participants and considerable proportions of participants being affected by other risk factors for contracting HIV and STIs.

### Knowledge about HIV and STIs and knowledge gaps among sub-groups

We identified sub-groups with particular knowledge gaps: i) migrants from sub-Saharan Africa of younger age, ii) recent migrants, iii) migrants from sub-Saharan Africa without regular access to the German health care system, iv) migrants from sub-Saharan Africa with lower socioeconomic status, and v) migrants from sub-Saharan Africa with Muslim religious affiliation.

We observed an association between younger age and lower levels of knowledge about HIV and STIs. This association is well described in previous studies conducted in sub-Saharan African countries and in the UK [37–41] and is common among the German general population as well [20]. Lower knowledge in younger age groups might be a result of the fact that knowledge accumulates with age. Lower German language proficiency was associated with lower knowledge about HIV in all three sections. If migrants do not bring this knowledge from their countries of origin, information about these topics for the general population might not be accessible for migrant groups, especially if language barriers exist [17]. Furthermore, shorter length of stay in Germany was associated with lower levels of knowledge regarding German HIV policies and HIV testing. And lower levels of knowledge were also found among those without regular access to the German health care system. This group includes asylum seekers who need a medical treatment voucher from social welfare offices and those with an undocumented legal status, whom we might have captured by the proxy of having no health insurance at all. Knowledge gaps might exist due to challenges other than HIV that may play a more important role in the lives of people having recently migrated to or seeking asylum in Germany, that is, their residence status or the need to find work. Especially for migrants with an undocumented legal status, the fear of deportation and the associated stress [42] can be an important barrier in seeking information about HIV prevention. Correlations between lower knowledge about HIV and STIs and lower levels of education and income are widely described in the literature [39, 41, 43–46] and were also found within our study. Information about HIV and STIs might be accessed and comprehended more easily by people with higher educational levels. For those with lower income, issues other than HIV and STIs might be the main focus of their lives. In addition, we found lower levels of knowledge about HIV and STIs among Muslim participants, similar to the UK [23]. Lack of knowledge about HIV and STIs remains a challenge that needs to be addressed in communities where sexual behavior is seen as a private matter that is not talked about, which often is the case in many Muslim communities [47, 48].

### Reaching sub-groups with information about HIV and STIs

The preferred methods of information dissemination among all participants were medical professionals and counseling centers. This supports the necessity of a smooth and efficient integration into the general health care system for all migrants irrespective of their health insurance status [49] to ensure the dissemination of important information [50,51] and linkage to testing and care [52,53]. Political solutions are necessary to tackle this issue, such as by granting full access to the health care system for asylum seekers, by prohibiting reporting to the foreigners’ registration offices when migrants without residence permit seek care, or by the introduction of anonymous medical cards given out by clearing houses and financed by emergency funds [54]. The second most often reported method of obtaining information about HIV and STIs was workshops. Offering these in shared accommodations for asylum seekers could be an effective way of knowledge dissemination, as recent migrants showed less knowledge. Workshops should be offered in different languages to also reach those with lower German language proficiency, who showed knowledge gaps within our study. Participants, especially younger ones, also wished to obtain information about HIV and STIs via new media, which might reflect their higher affection to new media. Younger participants showed less knowledge but less often expressed information needs, which might indicate a reduced perception of being at risk. However, by using new media, that is, *m*health channels for prevention they might be reached as well. Promising approaches of sending prevention messages regarding HIV and STIs on youths’ cell phones from Australia [55] or the US [56, 57] could be adapted for Germany, to address youth and particularly migrant youth. Although getting informed about HIV and STIs through the personal environment was less often stated by participants, future involvement of peer researchers in doing prevention work in their communities might be a useful way to reach those who do not actively seek information about HIV and STIs, such as through visiting workshops or counseling centers. In Germany there are already some model HIV prevention projects working with members of African communities [24, 58–60]. This could be put into general practice for HIV prevention with migrant communities. Additionally, discussing HIV in the communities increases the likelihood of getting tested [53]. The involvement of religious leaders in prevention planning might also be a promising approach to de-stigmatize topics related to HIV infections [47, 61]. A successful HIV prevention program under the involvement of pastors that is being established in African churches in Germany could be adapted and implemented in mosques [58, 60].

### Sexual behavior and other risk factors

Males showed higher levels of sexual risk behavior than females. These results are consistent with our findings from the pilot study [33] and with those from other European studies [22, 23, 62–64]. Comparable data on sexual risk behavior within the German general population are scarce. One study found more concurrent sexual contacts for males, although in this study the majority had reported consistent condom use with non-steady sexual partners (82%) [65].

Reasons for not using condoms differed between males and females, and our findings suggest difficulties in negotiating condom use for females, which might be a result of male dominance. This is supported by two reviews on studies conducted internationally and in sub-Saharan African countries, respectively, which found that gender inequality, gender norms, and associated difficulties to talk about sexuality make it challenging for women to insist on condom use [66, 67]. Studies have shown that the experience of sexualized violence, especially within partnerships, may further hinder women from insisting on condom use as they might fear further violence [68, 69].

Being affected by sexualized violence was reported by more than 10% of study participants. The occurrence of sexualized violence in armed conflicts is widely described [70, 71] and might have been the case for participants in our study, who reported to have experienced sexualized violence in their countries of origin. Moreover, inequalities, which might lead to dependencies within partnerships, may also lead to sexualized violence [69, 72]. This might be the case for those who reported to have experienced sexualized violence in Germany. The finding that male migrants from sub-Saharan Africa most often have had experienced sexualized violence in Germany should be further investigated.

Overall, our results call for gender-sensitive prevention strategies focused on the reduction of sexual risk behavior among males and empowerment for women in sexual relationships.

### Limitations

Some limitations have to be taken into account when interpreting our findings. Due to convenience sampling and uncertainties regarding the composition of African communities in Germany, our results might not be representative for all migrants from sub-Saharan Africa living in Germany, and there surely is a selection bias. Requirements for statistical measurements like OR may therefore not be completely fulfilled, and proportions as well as CI may reflect tendencies rather than statistical truth. However, with regards to gender and country of birth, our sample reflects well the official data [34]. We were able to include more migrants from sub-Saharan Africa with lower educational levels than was possible in other European studies [22, 23, 64]; thus, our results may include less educational bias. In terms of other sociodemographic characteristics, steering recruitment allowed us to obtain a heterogeneous sample.

Using true statements to capture knowledge about HIV and STIs may have led to an overestimation of knowledge. For us and our collaborating partners, it was more important to use the study as an intervention that increases knowledge, as migrants from sub-Saharan Africa taking part in the survey may not be reached by future interventions.

Social desirability bias may have led to an overestimation of knowledge, especially in interviews, and also might have influenced answers in the section on sexual behavior. We controlled for the mode of survey administration but did not find any evidence suggesting social desirability or information bias might have impacted the results. Recall bias might have influenced answers in the section on sexual behavior as well, and participants, for example, might have over- or underestimated the number of sexual partners they have had within the last 12 months. Another limiting factor in our study on sexual health is that we did not ask for sexual orientation, as our collaborating partners recommended us not to do so.

Another limiting factor was the availability of the questionnaire in only three languages, which might have led to exclusion of potential participants who did not want to answer the questions in a face-to-face or telephone interview but were not proficient in one of these languages.

## Conclusion

Utilizing CBPHR enabled us to identify knowledge gaps regarding HIV and STIs within a heterogeneous sample of migrants from sub-Saharan Africa living in six German cities and regions; additionally, we found sexual risk behavior especially among male participants and different risk factors for contracting HIV and STIs among female migrants from sub-Saharan Africa such as difficulties in negotiating condom use and sexualized violence. Groups with particular knowledge gaps were i) younger migrants from sub-Saharan Africa, ii) recent migrants, iii) those without regular access to the German health care system, iv) migrants from sub-Saharan Africa with lower socioeconomic status, and iv) those with Muslim religious affiliation. These groups should be addressed with targeted interventions, taking into account their preferred methods of information dissemination, that is, medical professionals and counseling centers. Young migrants from sub-Saharan Africa could be reached via *m*health channels. Male participants reported sexual risk behavior more frequently than females, and prevention messages for male migrants from sub-Saharan Africa should focus on their own risk perception. For female migrants from sub-Saharan Africa, prevention messages should focus on empowerment to enhance their ability to, for example, negotiate condom use. In the context of ongoing global migration, future prevention and intervention planning for migrants from sub-Saharan Africa living in Germany should consider the findings of our study and specifically target migrant groups most at risk.

## Supporting information

S1 Table Univariable analyses: Preferred methods of information dissemination stratified by sociodemographic characteristics of the participants of the misSA study in Germany, 2014–2016, n = 1,712. (DOC)
