# Supplementary material for: Knowledge, information needs and behavior regarding HIV and sexually transmitted infections among migrants from sub-Saharan Africa living in Germany: Results of a participatory health research survey
Source: PLoS One. 2020 Jan 27;15(1):e0227178. doi: 10.1371/journal.pone.0227178 (PMC6984683; doi:10.1371/journal.pone.0227178)
Supplement: S1 Table — (DOC) [file pone.0227178.s001.doc]

S1 Table. Univariable analyses: Preferred methods of information dissemination stratified by sociodemographic characteristics of the participants of the misSA study in Germany, 2014–2016, n=1,712

| **Methods of information dissemination** | | | | | |  |  |  |  |  |  |  |  |  |  |  |  |  |  |  |  |
| --- | --- | --- | --- | --- | --- | --- | --- | --- | --- | --- | --- | --- | --- | --- | --- | --- | --- | --- | --- | --- | --- |
| **Variable** | |  | **Health professionals** | | |  | **Workshops** | | |  | **New media** | | |  | **Classic and print media** | | | **%** | **Personal environment** | | |
|  | | **%** | **OR** | **95% - CI** | **p-value** | **%** | **OR** | **95% - CI** | **p-value** | **%** | **OR** | **95% - CI** | **p-value** | **%** | **OR** | **95% - CI** | **p-value** |  | **OR** | **95% - CI** | **p-value** |
| Sex | |  |  |  |  |  |  |  |  |  |  |  |  |  |  |  |  |  |  |  |  |
|  | Men (n=909) | 69.4% | Ref. |  |  | 48.7% | Ref. |  |  | 47.5% | Ref. |  |  | 42.8% | Ref. |  |  | 30.7% | Ref. |  |  |
|  | Women (n=803) | 73.5% | 1.22 | 0.98–1.51 | 0.064 | 52.7% | 1.17 | 0.97–1.42 | 0.104 | 47.7% | 1.01 | 0.83–1.22 | 0.944 | 36.9% | 0.78 | 0.64–0.95 | **0.012** | 27.8% | 0.87 | 0.70–1.07 | 0.185 |
| Age | |  |  |  |  |  |  |  |  |  |  |  |  |  |  |  |  |  |  |  |  |
|  | 18–25 years (n=354) | 67.8% | 0.59 | 0.43–0.81 | **0.001** | 47.7% | 0.74 | 0.56–0.98 | **0.035** | 55.4% | 1.63 | 1.23–2.16 | **0.001** | 39.0% | 0.95 | 0.72–1.27 | 0.741 | 34.5% | 1.30 | 0.96–1.75 | 0.087 |
|  | 26–35 years (n=603) | 67.3% | 0.58 | 0.44–0.77 | **<0.001** | 47.4% | 0.73 | 0.57–0.94 | **0.013** | 51.1% | 1.37 | 1.07–1.75 | **0.012** | 40.3% | 1.01 | 0.79–1.29 | 0.957 | 27.7% | 0.95 | 0.72–1.24 | 0.687 |
|  | 36–45 years (n=451) | 78.1% | Ref. |  |  | 55.2% | Ref. |  |  | 43.2% | Ref. |  |  | 40.1% | Ref. |  |  | 28.8% | Ref. |  |  |
|  | > 45 years (n=304) | 73.4% | 0.77 | 0.55–1.09 | 0.138 | 53.3% | 0.93 | 0.69–1.24 | 0.603 | 38.2% | 0.81 | 0.60–1.09 | 0.165 | 40.5% | 1.01 | 0.75–1.36 | 0.928 | 27.3% | 0.93 | 0.67–1.28 | 0.649 |
| Educational level | |  |  |  |  |  |  |  |  |  |  |  |  |  |  |  |  |  |  |  |  |
|  | No, primary, or secondary school (n=622) | 71.9% | 0.93 | 0.72–1.20 | 0.582 | 48.9% | 0.85 | 0.68–1.07 | 0.171 | 38.8% | 0.74 | 0.58–0.93 | **0.009** | 36.3% | 0.79 | 0.63–0.99 | **0.049** | 34.2% | 1.31 | 1.02–1.67 | **0.034** |
|  | High school/vocational School (n=558) | 73.3% | Ref. |  |  | 52.9% | Ref. |  |  | 46.2% | Ref. |  |  | 41.9% | Ref. |  |  | 28.5% | Ref. |  |  |
|  | University/college (n=532) | 68.6% | 0.80 | 0.61–1.03 | 0.088 | 50.2% | 0.90 | 0.71–1.14 | 0.376 | 59.4% | 1.70 | 1.34–2.16 | **<0.001** | 42.3% | 1.02 | 0.80–1.29 | 0.905 | 24.4% | 0.81 | 0.62–1.06 | 0.129 |
| Length of stay in Germany | |  |  |  |  |  |  |  |  |  |  |  |  |  |  |  |  |  |  |  |  |
|  | < 5 years (n=740) | 68.1% | 0.76 | 0.62–0.94 | **0.010** | 49.1% | 0.90 | 0.74–1.09 | 0.269 | 49.6% | 1.15 | 0.95–1.39 | 0.150 | 39.5% | 0.96 | 0.79–1.17 | 0.684 | 29.2% | 0.99 | 0.80–1.22 | 0.916 |
|  | ≥ 5 years (n=972) | 73.8% | Ref. |  |  | 51.8% | Ref. |  |  | 46.1% | Ref. |  |  | 40.4% | Ref. |  |  | 29.4% | Ref. |  |  |
| German language proficiency | |  |  |  |  |  |  |  |  |  |  |  |  |  |  |  |  |  |  |  |  |
|  | No, little, or unknown (n=483) | 69.6% | 0.89 | 0.70–1.14 | 0.356 | 47.6% | 0.80 | 0.64–1.01 | 0.057 | 46.8% | 1.01 | 0.81–1.27 | 0.905 | 38.9% | 1.00 | 0.80–1.26 | 0.984 | 30.2% | 1.04 | 0.81–1.32 | 0.776 |
|  | Average or good (n=831) | 72.0% | Ref. |  |  | 53.1% | Ref. |  |  | 46.5% | Ref. |  |  | 38.9% | Ref. |  |  | 29.5% | Ref. |  |  |
|  | Very good or mother tongue (n=398) | 72.1% | 1.01 | 0.77–1.31 | 0.957 | 49.0% | 0.85 | 0.67–1.08 | 0.181 | 51.0% | 1.20 | 0.95–1.52 | 0.135 | 43.7% | 1.22 | 0.96–1.56 | 0.105 | 27.9% | 0.93 | 0.71–1.21 | 0.565 |
| Monthly net income | |  |  |  |  |  |  |  |  |  |  |  |  |  |  |  |  |  |  |  |  |
|  | < 1,000 Euro per month (n=851) | 68.0% | 0.74 | 0.58–0.95 | **0.016** | 49.5% | 0.81 | 0.65–1.01 | 0.066 | 50.8% | 1.19 | 0.95–1.48 | 0.136 | 38.8% | 0.79 | 0.63–0.99 | **0.041** | 31.8% | 1.13 | 0.89–1.45 | 0.310 |
|  | ≥ 1,000 Euro per month (n=490) | 74.3% | Ref. |  |  | 54.7% | Ref. |  |  | 46.5% | Ref. |  |  | 44.5% | Ref. |  |  | 29.2% | Ref. |  |  |
|  | Unknown (n=371) | 74.9% | 1.03 | 0.76–1.41 | 0.829 | 47.7% | 0.76 | 0.58–0.99 | **0.042** | 41.8% | 0.83 | 0.63–1.08 | 0.165 | 36.9% | 0.73 | 0.55–0.96 | **0.026** | 23.7% | 0.76 | 0.55–1.03 | 0.074 |
| Health insurance status | |  |  |  |  |  |  |  |  |  |  |  |  |  |  |  |  |  |  |  |  |
|  | Regular health insurance (n=1,378) | 72.1% | Ref. |  |  | 51.3% | Ref. |  |  | 48.4% | Ref. |  |  | 41.4% | Ref. |  |  | 29.1% | Ref. |  |  |
|  | No health insurance or medical treatment voucher for asylum seekers or unknown (n=334) | 68.3% | 0.83 | 0.64–1.08 | 0.169 | 47.6% | 0.86 | 0.68–1.10 | 0.225 | 44.3% | 0.85 | 0.67–1.08 | 0.179 | 34.1% | 0.73 | 0.57–0.94 | **0.015** | 30.2% | 1.06 | 0.81–1.37 | 0.682 |
| Religious affiliation | |  |  |  |  |  |  |  |  |  |  |  |  |  |  |  |  |  |  |  |  |
|  | Christian (n=1,158) | 72.5% | Ref. |  |  | 50.7% | Ref. |  |  | 47.9% | Ref. |  |  | 40.9% | Ref. |  |  | 28.8% | Ref. |  |  |
|  | Muslim (n=432) | 69.2% | 0.85 | 0.67–1.08 | 0.191 | 51.4% | 1.03 | 0.82–1.28 | 0.804 | 45.1% | 0.89 | 0.72–1.12 | 0.322 | 35.7% | 0.80 | 0.64–1.01 | 0.059 | 31.5% | 1.14 | 0.90–1.45 | 0.289 |
|  | No, other, or unknown religion (n=122) | 67.2% | 0.78 | 0.52–1.16 | 0.214 | 46.7% | 0.85 | 0.59–1.24 | 0.405 | 53.3% | 1.24 | 0.85–1.80 | 0.261 | 47.5% | 1.31 | 0.90–1.91 | 0.154 | 27.1% | 0.92 | 0.60–1.40 | 0.691 |
